# Self-reported musculoskeletal disorder symptoms and associated factors among water carrying women in Legambo district, Northeastern Ethiopia: a community-based cross-sectional study design

**DOI:** 10.3389/fpubh.2024.1409535

**Published:** 2024-06-27

**Authors:** Gete Berihun, Kassahun Ayele Gasheya, Tadiwos Abebaw, Masresha Abebe, Adinew Gizeyiatu, Leykun Berhanu, Mengesha Dagne, Belay Desye, Zebader Walle, Leul Zewdu, Mebrat Gedfie Wondim

**Affiliations:** ^1^Department of Environmental Health, College of Medicine and Health Sciences, Wollo University, Dessie, Ethiopia; ^2^Department of Occupational Health and Safety, College of Medicine and Health Sciences, Wollo University, Dessie, Ethiopia; ^3^Department of Public Health, College of Health Sciences, Debre Tabor University, Debre Tabor, Ethiopia; ^4^Department of Special Needs and Inclusiveness, College of Social Sciences and Humanities, Debre Tabor University, Debre Tabor, Ethiopia

**Keywords:** water carrying practices, musculoskeletal disorders, women’s health, Legambo district, Northeastern Ethiopia

## Abstract

**Introduction:**

Musculoskeletal disorders are the leading cause of illness, disability, and poor quality of life. Lack of access to potable water in the backyard forces women to take water from off-plot sources every day, which may expose them to various health risks. However, there has been little investigation on the musculoskeletal disorders’ health effects on water-carrying women.

**Objective:**

This study aimed to assess musculoskeletal disorders symptoms and associated factors among water-carrying women in the Legambo district, Northeastern Ethiopia.

**Materials and methods:**

A community-based cross-sectional study was done with 618 water-carrying women chosen using simple random and systematic random sampling techniques. The data were collected using face-to-face interviews with the standard Nordic Musculoskeletal Questionnaire. Data entry were carried out using Epi-data version 4.6 and exported to SPSS version 25.0 for analysis. A binary logistic regression was used to determine the factors associated with self-reported musculoskeletal disorder symptoms at a 95% confidence interval (CI). In the multivariate model, variables with a *p*-value ≤0.05 and a 95% CI were declared as factors of self-reported musculoskeletal disorder symptoms. The model’s fitness was assessed using Hosmer and Lemeshow, and it was found to be fit.

**Results:**

The prevalence of self-reported MSD symptoms was 72.5% during the previous 12 months. MSD symptoms were significantly elevated among women who carried water from a distance of 501–1,000 m [adjusted odds ratio (AOR) = 5.39, 95% CI = 3.64–9.69] and >1,000 m (5.93, 2.84–12.40), carried a water load of >15 kg during pregnancy (8.29, 2.97–23.09), and carried a water load of >15 kg when not pregnant (1.59, 1.44–2.68).

**Conclusion:**

Three-fourths of the participants had self-reported musculoskeletal disorder symptoms in the past 12 months. Distance of water sources from their house, carrying the same amount of water during pregnancy, and weight of the water load carrying were factors associated with the self-reported musculoskeletal disorder symptoms. Hence, health professionals should raise awareness of the association between carrying high water loads and the development of MSDs, especially during pregnancy. Improvement in water supply infrastructure and enhancing behavioral intervention should be done. Furthermore, Future researchers should assess MSDs using objective measurements and cohort studies should be implemented.

## Introduction

Musculoskeletal disorders (MSDs) encompass a wide range of inflammatory and degenerative diseases affecting muscles, ligaments, tendons, nerves, bones, and joints (1–3). They are a major cause of illness in many countries, particularly developing countries, including Ethiopia. Work-related pain is becoming more prevalent, particularly among rural and disadvantaged people in developing countries (4). Workers in a variety of settings, including manufacturing, construction, agriculture, and transportation, who use their bare hands to operate tools, machines, and equipment in their daily lives are frequently subjected to uncomfortable postures, overextension, force exertion, and repetitive movements over long periods, which can result in serious injury or disability (5).

About one-quarter of the global population obtains their water from sources located away from their homes (6, 7). This off-premises water collection is common in low-income and middle-income regions where public water infrastructure is either inadequate or absent (8). As a result, women are often responsible for manually transporting large 20-liter containers of water, typically carrying them on their heads or other body parts (8, 9). More than two-thirds of the population in sub-Saharan Africa are obligated to leave their homes to collect water, and many rural water systems are often non-functional, exacerbating the difficulty of water collection and augmenting health problems (10). This physical water carriage can cause pain and mobility problems, which are key symptoms of musculoskeletal disorders (MSDs). These physical impacts may impair the quality of life and contribute to psychosocial stress for the women tasked with this labor (7, 9–11).

The Joint Monitoring Programme (JMP) report in 2017 revealed that 263 million people spent over 30 min per round trip to collect water from an improved water source (12). People who use unimproved or surface waters are more likely to take over 30 min to collect water (8). A study conducted in sub-Saharan African countries revealed that about 13.54 million women are responsible for household water collection taking more than 30 min per return trip in the region (10).

The impacts of fetching water on women’s health and ability to work are expected to be more pronounced in low- and middle-income nations where a larger number of people have engaged in physically demanding, informal, or poorly regulated work situations. Furthermore, economic, political, and social disparities are mirrored in access to drinking water. Furthermore, the negative economic and health effects of obtaining water are likely to disproportionately affect underprivileged groups (11). Despite water collection can hurt human health, only a few studies have been undertaken on its negative effects, such as weariness, musculoskeletal damage, and early degenerative bone and soft tissue injury (10). The most commonly reported adverse effect among the 39 water transporters in South Africa was spinal pain, at 69% (11). The problem is high in rural areas due to the presence of different confounding factors such as higher rates of poverty, chronic malnutrition, and poor health (10).

In 2017, 785 million people still lacked even a basic drinking water service (13). The problem is highly magnified in sub-Saharan Africa with half of the urban households getting their water sources outside of the home with the highest burden in rural areas reaching up to 89.5% (14). Despite different interventions being implemented to improve the accessibility of safe water supply, the problem still exists in all parts of the world with severe problems in developing countries including Ethiopia. The impacts of water carrying on MSDs are underreported which may be due to the little attention to date (6, 11). Hence, this study aimed to assess the self-reported MSDs symptoms and associated factors among water-carrying women in the Legambo district, Northeastern Ethiopia.

## Materials and methods

### Study area

The study was conducted in Legambo district which is located in South Wollo zone, Amhara Region, Ethiopia. The district has a total population of 189, 898 of which 178,420 were rural inhabitants whereas 11,478 were urban residents (15). The district has an area of 1017.35 km^2^ and an altitude range of 1,500–3,700 m. In Legambo district, there is one primary hospital, nine health centers, 10 private clinics, three private pharmacies, and 34 health posts (16). The district has 32 kebeles [the smallest administrative unit in Ethiopia (17)].

### Study design and period

A community-based cross-sectional study design was done to assess the prevalence of self-reported MSDs and associated factors among water carrier women in Legambo district, Northeastern Ethiopia. The study was conducted from July 1–30, 2022.

### Source and study population

The source population of the study was women in the Legambo district, South Wollo zone, Northeastern Ethiopia. On the other hand, women who lived in randomly selected eight kebeles of Legambo district, Northeastern Ethiopia were considered as the study population.

### Eligibility criteria

#### Inclusion criteria

The inclusion criteria were (1) having lived in the study location for at least 12 months; (2) ≥18 years of age; (3) having <5 children; and (4) providing informed consent to participate in the study.

#### Exclusion criteria

The exclusion criteria were (1) women with non-regular water carrying women; (2) used non-human means of water carrying technique; (3) those with a serious medical illness during the data collection; (4) had a history of previous musculoskeletal surgery; and (5) obvious skeletal deformities.

### Sample size estimation and sampling procedures

The sample size was estimated using a single population proportion formula taking the assumptions of the desired level of confidence (95% confidence interval), an acceptable margin of error (0.05), and the design effect of the sampling technique (1.5), the estimated proportion of self-reported MSDs symptoms (50%), and 10% non-response rate which gives the sample size of 633. A mixture of multistage sampling, simple random sampling, and systematic sampling techniques was employed to select participants in the study. Initially, 8 kebeles were selected from a total of 32 kebeles by a simple random sampling technique using a lottery method. Then, the households from the selected kebeles were selected using a systematic random sampling technique which was obtained from the health posts of the respective kebeles. Then, proportional allocations based on the eligible participants were carried out to determine the number of participants in each kebele.

### Outcome variable

The outcome variable of the study was self-reported musculoskeletal disorder symptoms with the option either (yes/no).

### Operational definitions

#### Self-reported MSDs symptoms

It is defined as a self-report of one or more body parts having pain, numbness, tingling, aching, stiffness, or burning, Pain continued in any part of body segments (neck, shoulders, upper back, lower back, elbows, wrists/hands, thighs/hips, knees, and ankles) caused, aggravated or exacerbated by workplace exposures pain lasting >3 days during the period for the past 12 months (18–22).

### Data collection tools and techniques

Data were collected using a structured questionnaire based on the Standardized Nordic Questionnaire (SNQ). The questionnaire was initially written in English, then translated into the local language (Amharic), and finally returned to English to ensure its consistency. The questionnaire has four sections: Part I: (socio-demographic characteristics of the respondents); Part II: (pregnancy-related conditions); Part III: water-carrying practice; and Part IV: self-reported MSD symptoms in the last 12 months. The presence of self-reported MSD symptoms over the previous 12 months was evaluated. The weight of the load, the weight of the ladies, and the height of the participants were all assessed, and their BMI was determined by dividing the weight in kg by height square in meters. Beam balance was used to measure the weight of the load and the weight of the women. Data were collected using a combination of face-to-face interviews, observation checklists, and physical measurements. The data collection was carried out by four individuals who held Bachelor of Science (BSc) degrees in Nursing and supervised by experts in human anatomy. This data collection took place from July 1 to July 30, 2022.

### Data quality assurance

The Data quality were ensured through different measures. A standardized questionnaire was used for data collection. Before data collection, the data collectors and supervisors were given 2 days of training. The contents of the training include the study objectives, content of the questionnaire, data collection procedures, and other relevant issues. Additionally, a pre-test was conducted on 5% of the sample size in the Dessie Zuria district and necessary amendments were carried out based on its feedback. During the final data collection, daily supervision was carried out and immediate correction was done for incomplete or missing data. Furthermore, a quality check was performed on 10% of the entered data to identify and address any data entry errors.

### Data management and analysis

Data entry and analysis were carried out using Epi-data version 4.6 and SPSS version 25, respectively. Descriptive statistics were employed to present the findings. A binary logistic regression model with a 95% CI was used to investigate the association between the predictor and outcome variables of the study. Initially, in bi-variable analysis variables with a *p*-value <0.25 were retained for multivariable logistic regression analysis. Then, in a multivariate analysis, variables with a *p*-value <0.05 were declared as factors significantly associated with the prevalence of self-reported MSDs among water-carrying women. The multi-collinearity among independent variables were also assessed using standard error at a cutoff value of −2 to +2. The model’s fitness was assessed using the Hosmer–Lemeshow test and it was fit.

### Ethics approval

The ethical approval was obtained from Wollo University’s College of Medicine and Health Sciences’ research and ethical committee under the reference number CMHS/526/20/14. Permission was received from the Legambo district administration. Following an explanation of the research purpose, participants who could read and write gave written consent, whereas those who could not verbal consent. The confidentiality of the study participants was assured by avoiding potential identifiers such as their names. Participants were also advised that they might refuse, withdraw, or completely reject their participation in the study. Finally, participants were informed that there were no incentives to engage in this study.

## Results

### Socio-demographic characteristics of the respondents

Table 1 presents the socio-demographic characteristics of the participants; a total of 618 women participated in the study with a response rate of 97.6%. The mean age of the participants was 33 ± 7 (mean with standard deviation). One-third of the participants were unable to read and write and two-thirds (68.6%) lived their lives through agricultural activities. Two-thirds (67%) of the study participants were underweight (<18.5). More than three-quarters (83.5%) of the participants visited healthcare facilities in the past year.

**Table 1:** Socio-demographic characteristics of the water-carrying women in Legambo district South Wollo zone, Northeastern Ethiopia in 2022.

Variable	Category	Frequency	Percentage
Age (years)	18–30	247	40
	31 and above	371	60
Religion	Orthodox	202	32.7
	Muslim	374	60.5
	Protestant	42	6.8
Marital status	Single	37	6.0
	Married	449	72.7
	Divorced	69	11.2
	Widowed	63	10.2
Educational status	Unable to read and write	215	34.8
	Informal education	228	36.9
	Primary education	112	18.1
	Secondary education	63	10.2
Occupation	Farmer	424	68.6
	Others	194	31.4
BMI index	Underweight (<18.5)	414	67.0
	Normal (18.5–24.9)	214	33.0
Self-reported health status	Good	151	24.4
	Medium	294	47.6
	Poor	173	28.0
Frequency of physician visits in the past year	None	103	16.7
	One	188	30.4
	Two	217	35.1
	Three and above	110	17.8
The presence of children in households	Yes	543	87.9
	No	75	12.1

### Pregnancy-related variables

Table 2 presents and pregnancy-related variables of the participants; nearly three-forth (71.2%) of the respondents had four and above parity. More than half of 300 (55.2%) of the respondents had their first childbirth at the age of less than 18 years old. On the other hand, less than 10 % 45 (7.3%) of the respondents had a history of abortion in their lifetime. Additionally, less than a quarter of the respondents 97 (15.7%) were pregnant during the survey time. Of these, about (10.3%) of the respondents were in the third-trimester stage.

**Table 2:** Pregnancy-related variables of the water-carrying women in Legambo district, South Wollo zone, Northeastern Ethiopia in 2022.

Variable	Category	Frequency (*n*)	Percentage (%)
Parity of mothers	None	75	12.1
	1–3	103	16.7
	≥4	440	71.2
Pregnancy	None	75	12.1
	1–2	30	4.9
	3–4	289	46.8
	≥5	224	36.2
Age at first childbirth (in years)	13–19 Years	300	55.2
	≥20 Years	243	44.8
Time since last birth (in months)	Less than six months	100	18.4
	6–12 months	218	40.1
	Greater than 12 months	225	41.4
Do you have a history of abortion	Yes	45	8.3
	No	489	91.7
Are you current pregnancy	Yes	97	15.7
	No	521	84.3
Gestational age	First trimester	42	43.3
	Send trimester	45	46.4
	Third trimester	10	10.3
Have you received help in carrying water	Yes	236	38.2
	No	382	61.8

### Water-carrying related factors

Table 3 presents water-carrying related factors of the participants; nearly, two-thirds of 397 (63.6%) of the respondent’s main sources of water supply were located within a 500-meter distance from their house. Three out of five, 372 (60.2%) of the women carry >15 kg of water from the source. Half 284 (52.3%) of the women reported that they had a habit of water carrying during pregnancy with approximately similar load in the absence of pregnancy time. Nearly half of the women carry water two times daily. Furthermore, one-third 205 (33.2%) of the respondents practiced keeping their backs straight. On the other hand, the majority of the respondents 251 (406%) applied Bending knees in the lifting of the water sources.

**Table 3:** Water carrying and related conditions among water-carrying women in Legambo district, South Wollo zone, Northeastern Ethiopia in 2022.

Variable	Category	Frequency (*n*)	Percentage (%)
Distance of main water source from the house (in meters)	0–500 m	393	63.6
	501–1,000 m	67	10.8
	Greater than 1,000 m	158	25.6
Time taken for one trip (in minutes)	<30 min	408	66.0
	≥30 min	210	34.0
Weight category (in kg)	≤15 Kg	246	39.8
	>15 kg	372	60.2
Carry the same amount of load during pregnancy	Yes	284	52.3
	I carry less	185	34.1
	I do not carry	74	13.6
Carry the same amount of load 3 months after delivery	Yes	337	62.1
	I carry less	181	33.3
	I do not carry	25	4.6
Frequency of water collection per day (in number)	1 time	109	17.6
	2 times	289	46.8
	3 times	192	31.1
	4 and above times	28	4.5
Dominant hand	Left	28	95.5
	Right	590	4.5
Frequency of physician visits in the past year	None	103	16.7
	One time	188	30.4
	Two times	217	35.1
	Three times and above	110	17.8
Water lifting technique	Keeping the back straight	205	33.2
	Bending knees	251	40.6
	Twisting	162	26.2

### Self-reported MSDs symptoms

Table 4 presents the self-reported MSDs symptoms of the participants; more than two-thirds of 422 (68.3%) of the respondents reported that they had pain or discomfort in the neck during the past year. Additionally, an almost similar number of the participants 420 (68.0%) reported they had pain or discomfort in their lower backs during the past year. On the other hand, nearly two-thirds of 392 (63.4%) of the study participants had pain or discomfort in the shoulders. Generally, this finding revealed that the prevalence of self-reported MSDs symptoms among water-carrying women was 448 (72.5%).

**Table 4:** Prevalence of self-reported musculoskeletal disorder symptoms in different parts of the body among water-carrying women in Legambo district, South Wollo zone, Northeastern Ethiopia in 2022.

Variable	Category	Frequency	Percentage
Have you had pain or discomfort in your neck during the past year	No	196	31.7
	Yes	422	68.3
Have you had pain or discomfort in your upper back during the past year	No	198	32.0
	Yes	420	68.0
Have you had pain or discomfort in your lower back during the past year	No	236	38.2
	Yes	382	61.8
Have you had pain or discomfort in your shoulders during the past year	No	226	36.6
	Yes	392	63.4
Have you had pain or discomfort in your Biceps during the past year	Yes	173	28.0
	No	445	72.0
Have you had pain or discomfort in your triceps during the past year	No	211	34.1
	Yes	407	65.9
Have you had pain or discomfort in your elbow during the past 1 year	Yes	138	22.3
	No	480	77.7
Have you had pain or discomfort of your lower arms during the past year	No	202	32.7
	Yes	416	67.3
Have you had pain or discomfort of your wrist during the past year	No	238	38.5
	Yes	380	61.5
Have you had pain or discomfort of your hand during the past year	No	214	34.6
	Yes	404	65.4
Overall self-reported MSDS symptoms	Yes	170	27.5
	No	448	72.5

### Factors associated with self-reported MSDs symptoms

Table 5 presents factors associated with self-reported MSDs symptoms of the participants; in multivariable logistic regression analysis, the distance of the main source of water, weight of the load carrying, and carrying the same amount of load during pregnancy were factors of self-reported MSDs symptoms among water-carrying women. Those who carried water from 501 to 1,000 m and above 1,000 m distance were AOR = 5.39 (95% CI, 3.64, 9.69) and AOR = 5.93, (95%CI, 2.84, 12.40) times more likely to have self-reported MSDs symptoms than those who carried within a distance of 500 m, respectively. Furthermore, women who carried the same amount of water load during pregnancy were AOR = 8.29 (95%CI, 2.97, 23.09) times more likely to have self-reported MSDs symptoms than those who did not carry water during their pregnancy. Finally, women who carried water more than 15 kg were AOR = 1.59, (95%CI, 1.44, 2.68) times more likely to have a self-reported MSDs symptom than those who carried less than 15 kg.

**Table 5:** Factors associated with self-reported musculoskeletal disorder symptoms among water carrier women in Legambo district, South Wollo zone, Northeastern Ethiopia in 2022.

Variables	Category	Self-reported MSDs		COR (95% CI)	AOR (95% CI)	*P*-value
		Yes	No			
Self-reported heath status	Good	79	72	Ref	Ref	
	Medium	219	75	2.66 (1.76–4.02)	0.99 (0.48–2.06)	0.986
	Poor	150	23	5.99 (3.45–10.23)	1.22 (0.51–2.92)	0.650
Number of parity	None	35	40	Ref	Ref	
	1–3	79	24	3.76 (1.98–7.16)	0.75 (0.38–1.50)	0.420
	≥4	334	106	3.60(2.18–5.96)		
The presence of children in the house	Yes	413	130	3.63 (2.21–5.95)	0.94 (0.56–1.59)	0.829
	No	35	40	Ref	Ref	
Age at first child (years)	≤18	239	61	0.64 (0.43–0.96)	1.12 (0.67–1.87)	0.659
	>18	174	69	Ref	Ref	
Received help from other family members in carrying water	Yes	135	101	Ref	Ref	
	No	313	69	3.39 (2.35–4.90)	0.94 (0.56–1.59)	0.829
Distance of main water source from house	0–500	243	150	Ref	Ref	
	501–1,000	63	4	9.72 (3.47–27.26)	5.38 (3.64–9.69)	<0.001*
	>1,000	142	16	5.48 (3.14–9.55)	5.93 (2.84–12.40)	<0.001*
Water lifting technique	Keeping the back straight	179	26	Ref	Ref	
	Bending knees	184	67	6.24 (3.73–10.43)	1.54 (0.77–3.08)	0.221
	Twisting	85	77	2.45 (1.64–3.77)	0.64 (0.35–1.18)	0.152
Weight of the load	≤15 Kg	143	103	Ref	Ref	
	>15 kg	305	67	3.28 (2.27–4.73)	1.59 (1.44–2.68)	0.010*
Did you carry the same amount of load during pregnancy	Yes	214	70	0.86 (0.56–1.31)	1.96 (1.15–3.35)	0.140
	Yes I carry less	134	51	4.51 (1.75–11.64)	8.29 (2.97–23.09)	<0.001*
	I’d not carry	69	5	Ref	Ref	

AOR, adjusted odd ratio; COR, crude odd ratio. Ref, reference variables; ^*^Refers to factors significantly associated with outcome variables.

## Discussion

The MSDs cause an enormous global disease burden with the highest disability and the fourth highest burden of all non-communicable diseases, particularly in developing countries including Ethiopia. However, there are still major gaps in the understanding of the prevalence of the problem and its determinant factors (20). Based on the WHO report in 2019, the MSDs was the leading cause of disabilities worldwide and accounted for the greatest proportion of loss of productivity at the workplace (22). This finding revealed that nearly three-quarters (72.5%) of the participants had self-reported MSDs symptoms in the past 12 months. Distance of main sources of water supply from home, carrying the same amount of water load during the pregnancy, and weight of water load carrying were factors of self-reported MSDs symptoms among participants of the study.

This finding indicated that the overall prevalence of self-reported MSDs symptoms among water-carrying women was 72.5% which was consistent with the study conducted in Brazil (72.7%) (23). However, this result was greater than that of studies done in Ethiopia with healthcare professionals 64.2% (24), 40.1% (25), hairdressers (47.5%) (26), computer users (bankers) (58.8%) (27), and healthcare providers (44.2%) (28), diabetic patients (23.29%) (29), barber workers (55.7%) (30), and cleaners (52.3%) (31).

On the contrary, this finding was lower than the studies conducted among teachers in Chile (88.9%) (32), University teaching staff in Cameroon (80.8%) (22), cleaners of health institutions in Ethiopia (83.1%) (33), school teachers in Chuquisaca, Bolivia 86% (34), heavy load carriers in Yaoundé city, Cameroon 100% (35), restaurant Workers in Ethiopia (81.5%) (36). The possible reason for this variation might be due to the variation in water-carrying practices in different parts of the world such as carrying on the head, on the waist, and the variation in water containers using either a basket or straps tied around the head to carry water containers (20).

Off-plot access to water commonly requires a household member to complete multiple water-collecting trips to meet the needs of the household consumption (13, 37). This finding revealed that more than a quarter of the respondents were obligated to travel to a minimum distance of 1,000 m to reach the main sources of water for a single trip which is a useful indicator of exposure time to sustained compressive loading (11). The burden of water carrying depends not only on its distance but also on other factors such as walking to a source, joining long queues, filling containers, and carrying them home and environmental factors, particularly the incline or gradient of the path along which water is carried are likely to influence the physical work of water carrying (11, 38). People with the problem of MSDs may experience difficulty in carrying water and are obliged to seek help from others. This problem affects the family members in terms of a reduction in the usual volumes of water collected to support health and adequate hygiene (11). The practice of water carrying is one of the known risk factors for the development of MSDs (6, 14). Therefore, women who are responsible for these tasks are highly vulnerable to such types of problems, especially in low and middle-income countries (13).

In Ethiopia, only 50% of rural and urban households have access to essential water services, defined as water from an improved source with a collection time of no more than 30 min round trip (39). The weight load should be in the range of 10 to 15 kg to prevent the occurrences of MSDs (20). This finding showed that nearly two-thirds (60.2%) of the participants carried greater than 15 kg which is a lead for the occurrences of MSDs symptoms. This finding revealed that the weight of the water container was one of the factors that affect the prevalence of MSDs which was matched with other findings (11, 21). Furthermore, the maximum recommended backpack weights should be limited to 10–15% of body weight or less. However, this finding revealed that the ratio reaches up to 50% and above which is 5 times higher than the recommended level. Hence, frequent exposure to such amounts of unbalanced load may lead to increments of the problem (11). The problems may be worsening during the dry season since this study was conducted during the rainy season which has an alternate source of water supply (20).

The recommended maximum loads during the first trimester should be within the range of 7–11 kg and should not be lifted by themselves from the ground. But in this finding, almost all women carried the load by lifting from the ground which contrasts with the standard guideline with frequent practices in the likelihood of different health risks, MSDs. The problem may also result in a three-times-higher risk of preterm labor and miscarriages. Furthermore, it is also recommended that no loads heavier than the baby should be carried until 6 weeks postpartum and no “very heavy loads should be carried until 3 months postpartum”. However, most of the women in low and middle-income countries do not meet this strategy, including the findings of this study (6). The finding of the current study revealed that most of the women during pregnancy and 3 months after delivery carried even an equal load with non-pregnant women was another factor of MSDs among the participants of the study (40). This study’s overall implications could aid in the development of interventions, improve the quality of life for these populations, advance gender equality, and contribute to the body of knowledge regarding the relationship between gender, labor, and health.

### Limitations of the study

This study has a few drawbacks. The data were cross-sectional, thus we cannot draw causal conclusions. Additionally, MSDs symptoms were assessed using respondents’ self-reported data, which may be prone to social desirability bias.

## Conclusion

In conclusion, over three-quarters of the participants reported MSDS symptoms within the previous year. The distance between water sources and their homes, the practice of carrying the same amount of water during pregnancy, and the weight of the load carried were factors significantly associated with self-reported MSDS symptoms among water-carrying women. Hence, improvements to water supply infrastructure, promotion of intermediary solutions such as carts, bicycles, and self-supply options, particularly for women living in hilly areas, and interventions on behavioral modification should be done. Future research should use objective measurement of diagnosing MSDs diagnosis using a cohort study design. Future research should investigate the association between the intensity, frequency, and duration of water loading and the development of MSDs.

## Data availability statement

The raw data supporting the conclusions of this article will be made available by the authors, without undue reservation.

## Author contributions

GB: Conceptualization, Formal analysis, Funding acquisition, Investigation, Methodology, Project administration, Resources, Software, Supervision, Validation, Visualization, Writing – original draft, Writing – review & editing. KG: Conceptualization, Formal analysis, Investigation, Methodology, Software, Supervision, Validation, Visualization, Writing – original draft, Writing – review & editing. TA: Software, Supervision, Validation, Visualization, Writing – review & editing. MA: Formal analysis, Methodology, Supervision, Writing – review & editing. AG: Conceptualization, Software, Supervision, Validation, Writing – review & editing. LB: Conceptualization, Methodology, Supervision, Writing – review & editing. MD: Formal analysis, Software, Supervision, Validation, Writing – review & editing. BD: Supervision, Writing – original draft, Writing – review & editing. ZW: Formal analysis, Methodology, Validation, Writing – original draft, Writing – review & editing. LZ: Supervision, Visualization, Writing – review & editing. MW: Supervision, Validation, Visualization, Writing – review & editing.
